# PEG-BCT-100 and Canavanine Synergistically Induce Apoptosis in Arginine Biosynthetic Enzyme-Deficient Pancreatic Cancer

**DOI:** 10.1158/2767-9764.CRC-24-0425

**Published:** 2024-12-23

**Authors:** Tsz Tung Kwong, Hao Hao Deng, Chi-Hang Wong, Anthony W.H. Chan, Landon Long Chan, Siu-Ho K. Chok, Paul N. Cheng, Stephen Chan

**Affiliations:** 1 Department of Clinical Oncology, Faculty of Medicine, The Chinese University of Hong Kong, Hong Kong SAR, China.; 2 Department of Anatomical and Cellular Pathology, Faculty of Medicine, The Chinese University of Hong Kong, Hong Kong SAR, China.; 3 Department of Surgery, Faculty of Medicine, The Chinese University of Hong Kong, Hong Kong SAR, China.; 4 BioCancer Treatment International Ltd, Hong Kong SAR, China.

## Abstract

Arginine deprivation using arginase emerges as a potential cancer treatment approach, as some cancers have been found to be arginine-auxotrophic, relying on exogenous arginine for their growth. In this study, we evaluated the combination of a PEGylated recombinant human arginase (PEG-BCT-100), which is currently undergoing clinical trials, with a toxic arginine analog, canavanine, in pancreatic cancer. Remarkable synergistic antitumor effect was observed both *in vitro* and *in vivo*. Specifically, the combination treatment induced apoptotic cell death in pancreatic cancer cells, whereas normal fibroblast cells remained viable. Our findings also suggested that pancreatic tumors lacking the key enzymes in arginine biosynthesis, argininosuccinate synthetase 1 (ASS1) and ornithine transcarbamylase (OTC), were more susceptible to the treatment. From our in-house cohort and online database analysis, we found that the majority of the patients with pancreatic cancer exhibited deficiencies in both ASS1 and OTC enzymes, suggesting that the combination of arginine deprivation and canavanine could be particularly effective in these patients. The ASS1 and OTC negativity could also serve as predictive biomarkers for the response in other arginine-dependent cancers.

**Significance:**

This study investigates the synergistic antitumor effect of PEG-BCT-100, an arginase, in clinical trials, with canavanine in pancreatic cancer, *in vitro* and *in vivo*. The treatment induces cancer cell apoptosis while sparing normal fibroblasts. Our findings suggest heightened susceptibility of pancreatic tumors deficient in arginine biosynthesis enzymes ASS1 and OTC.

## Introduction

Pancreatic cancer is the leading cause of cancer-related deaths in developed countries, with the lowest 5-year survival rate among all cancers at 12% (1, 2). More than 80% of cases are diagnosed at an advanced stage, either locally advanced or metastatic, rendering the tumors unresectable (3). Currently, chemotherapy serves as the standard therapy for metastatic pancreatic cancer. Despite an increasing number of approved chemotherapy agents or regimens, the median overall survival for patients with metastatic pancreatic cancer remained less than 12 months (2, 4, 5). Therefore, there is a pressing need for novel treatment alternatives for advanced pancreatic cancer.

Arginine deprivation has emerged as a potential anticancer treatment due to the physiologic vital role of arginine in cell growth and proliferation. Arginine, a semi-essential amino acid, serves as a precursor for the biosynthesis of various essential compounds, including proteins, polyamines, creatinine, and nitric oxide (ref. 6). Arginine-derived nitric oxide is found to be associated with tumorigenesis and invasion (7). Given the involvement of arginine in diverse biological pathways, therapeutic approaches using enzyme-mediated arginine deprivation, such as arginase, have been explored. In this study, a clinical-grade PEGylated recombinant human arginase I (PEG-BCT-100) with enhanced catabolic activity and an extended *in vivo* half-life was utilized. Previous human clinical trials reported no immunogenicity or toxic effects from the end products of metabolism (8, 9). Furthermore, our group completed a phase II trial (NCT01092091), demonstrating tolerability and a modest disease control rate for hepatocellular carcinoma with weekly i.v. injections of PEG-BCT-100 at 2.7 mg/kg (10). The sensitivity to arginine deprivation is associated with the availability of arginine. Therefore, tumors that lack or have low expression of arginine *de novo* synthesis enzymes, such as argininosuccinate synthase 1 (ASS1) and ornithine transcarbamylase (OTC), are postulated to be more susceptible to arginase treatment. Despite ASS1 being universally expressed as a urea cycle enzyme in almost all cell types, various cancer types, including pancreatic carcinoma, are found to be ASS1-deficient (11–13). This highlights the rationale for utilizing arginase as a treatment option for pancreatic cancer.

Canavanine is a toxic analog of arginine which can be isolated from leguminous plants (14). Due to its structural similarity to arginine, canavanine can be incorporated into newly synthesized proteins, resulting in the rapid disruption of the functionality of these canavanine-containing proteins. Ultimately, critical reactions involved in RNA and DNA metabolism, as well as protein synthesis, are impaired (14, 15). Canavanine has demonstrated antineoplastic activity against various cancer cell line models (16, 17). Previous studies have shown that canavanine can sensitize the response to radiotherapy and exhibit cytotoxic effect on pancreatic cells, albeit at a relatively high dosage in the millimolar (mmol/L) range when used as a monotherapy (18, 19). By depleting arginine through the clinically safe arginase, PEG-BCT-100, we propose that the uptake of toxic canavanine could be facilitated, potentially leading to synergistic antitumorigenic effects in pancreatic cancer while reducing the general toxicity associated with canavanine. Additionally, we evaluated the expression levels of ASS1 and OTC in pancreatic cancer and their correlation with the efficacy of the combination treatment.

## Materials and Methods

### Cell lines and reagents

Human pancreatic cancer cell lines MIA PaCa-2 (RRID: CVCL_0428) and CFPAC-1 (RRID: CVCL_1119) and human lung fibroblast cell line WI-38 were purchased from the ATCC. All cell lines were cultured in high-glucose DMEM (HyClone) with 10% FBS (HyClone) and 1% penicillin/streptomycin (GIBCO) in a humidified incubator with 5% CO_2_ at 37°C. The WI-38 cell line (RRID: CVCL_0579) was supplemented with 1% minimum essential medium nonessential amino acids (GIBCO) for no more than 20 passages. Cell lines were subjected to *Mycoplasma* testing (MycoAlert, Lonza Bioscience) prior to experiments. PEG-BCT-100 was provided by Bio-Cancer Treatment International Ltd, and canavanine was purchased from Sigma-Aldrich (C1625).

### Cytotoxicity assay

Cells were seeded in 96-well plate and allowed to adhere for 24 hours. Cells were treated with canavanine at concentrations ranging from 1 pmol/L to 20 mmol/L in either complete DMEM or arginine-free DMEM and then incubated for various time points. Cell viability was quantified using WST-1 assay reagent (Abcam) following the manufacturer’s protocol. Absorbance was measured at 450/690 nm using a microplate reader (PerkinElmer 1420 Multilabel Counter VICTOR3). Cell growth inhibition was normalized to the untreated control, and the IC_50_ value was calculated.

### Live-cell time-lapse imaging

Cells were plated with 3 × 10^4^ cells per well for MIA PaCa-2 and WI-38 and 5 × 10^4^ cells per well for CFPAC-1. For coculture experiment, WI-38 and the stable GFP-expressing MIA PaCa-2 cells were seeded onto the same well accordingly. After 24 hours, the cells were administrated with PEG-BCT-100 alone (0.3 IU/mL) or in combination with canavanine (10 or 50 μmol/L) for 96 hours with or without the addition of urea cycle enzyme substrates (1 mmol/L citrulline and 1 mmol/L ornithine) before time-lapse imaging. Images were captured every 2 hours using Axio Observer Z1 (Carl Zeiss) equipped with a live-cell chamber (37°C, 5% CO_2_, and 95% humidity). Cell viability was detected using LIVE/DEAD Viability/Cytotoxicity Kit (Invitrogen) and visualized under a fluorescence microscope. The area and fluorescence intensity were quantified by ImageJ software.

### Annexin V/propidium iodide apoptosis assay

Apoptotic events upon drug treatment were determined using Annexin-V-Fluos (Roche Applied Sciences) according to the manufacturer’s instructions. The cells were washed twice with PBS and stained with 100 μL mixture of Annexin-V-Fluos (1:50), propidium iodide (PI; 1 μg/mL), and RNase (5 μg/mL) in 1X annexin V–binding buffer (10 mmol/L HEPES/NaOH, pH 7.4, 140 mmol/L NaCl, and 5 mmol/L CaCl_2_) for 15 minutes at room temperature in the dark. The samples were analyzed by flow cytometry (BD Biosciences, BD Accuri C6), and both early (annexin V–positive/ PI-negative) and late (double-positive) apoptotic events were included for cell death determination.

### Cell death ELISA assay

Cells were seeded at a density of 3,000 cells per well in a 96-well plate. Following drug treatment, cell death was quantified using Cell Death Detection ELISA^PLUS^ Kit (Roche Applied Sciences). Absorbance at 405 nm was measured with a reference wavelength of 490 nm using a microplate reader (PerkinElmer 1420 Multilabel Counter VICTOR3).

### Western blotting

Whole-cell lysates were prepared using RIPA lysis buffer (Thermo Fisher Scientific) containing a cocktail of protease and phosphatase inhibitors. Proteins concentrations were quantified via the bicinchoninic acid assay. Equal amounts of proteins were resolved on SDS-PAGE gels and transferred onto a polyvinylidene difluoride membrane and blocked with 5% nonfat dry milk. The following specific primary antibodies were used: ASS1 (PA5-21351, Thermo Fisher Scientific; RRID: AB_11153096) and OTC (PA5-28197, Thermo Fisher Scientific; RRID: AB_2545673), and Apoptosis Antibody Sampler Kit (#9915, Cell Signaling Technology) was used. Following the incubation with horseradish peroxidase–conjugated secondary antibodies, the blots were developed with enhanced chemiluminescence substrate (GE Healthcare Life Sciences) by autoradiography.

### *In vivo* efficacy studies on a MIA PaCa-2 xenograft model

Three- to four-week-old female athymic nude mice (nu/nu) were used in this study with the approval of the Animal Experimentation Ethics Committee and supplied by the Laboratory Animal Services Center at the Chinese University of Hong Kong (CUHK). S.c. xenografts were established in the bilateral flanks of nude mice by the inoculation of 2 × 10^6^ cells in Hank’s Buffered Salt Solution. When tumors reached 3 to 5 mm diameter, mice were randomized into different groups as follows: vehicle control, PEG-BCT-100 (10 mg/kg, twice per week), canavanine (500 mg/kg, daily), and the combination of PEG-BCT-100 and canavanine. Drugs were diluted with a vehicle (PBS) and administrated by i.p. injection in a volume of 200 μL. Canavanine was delivered daily, whereas PEG-BCT-100 was given twice per week following a 7-day schedule. The first dose of canavanine was administered 2 days after PEG-BCT-100 to allow for arginine depletion first. If two drugs were given on the same day, canavanine was administered 6 hours later to PEG-BCT-100 in the mice. The size of tumor nodules was measured with a caliper at least twice a week until euthanization.

### Pancreatic patient samples

Sections of formalin-fixed paraffin-embedded tumor tissues were acquired from 61 pancreatic cancer cases at the Prince of Wales Hospital, Hong Kong, with written informed consent obtained from the patients under the guideline from the Joint The Chinese University of Hong Kong (CUHK)-New Territories East Cluster (NTEC) Clinical Research Ethics Committee. The expression of ASS1 and OTC was assessed using the Histoscore (H-score) by the pathologist from the Department of Anatomical and Cellular Pathology, CUHK, as previously described (20). The H-score provides a semiquantitative classification system for IHC staining intensity, with the score ranging from 0 (100% of cells being negative) to 300 (100% of cells being strongly positive).

### Public dataset analysis

The Cancer Genome Atlas (TCGA) contains functional genomic datasets of different tumors, whereas Human Protein Atlas (HPA) is a database exploring the human proteins in cells, tissues, and organs levels (21, 22). The pan-cancer analysis of ASS1 and OTC was conducted using data extracted from TCGA and HPA.

### Statistical analysis

Analyses were performed using GraphPad Prism software version 10.0 (PRISM10; GraphPad Software Inc.). Statistical analysis was performed with one-way ANOVA. Analysis of tumor volume was performed with unpaired *t* test. The results were considered statistically significant when *P* value < 0.05 and denoted by *.

### Data availability

Data are available upon request from the corresponding author. The data analyzed in this study are available from TCGA and HPA.

## Results

### Arginine deprivation by PEG-BCT-100 synergizes with canavanine in pancreatic cancer cell growth inhibition

**Figure 1:**
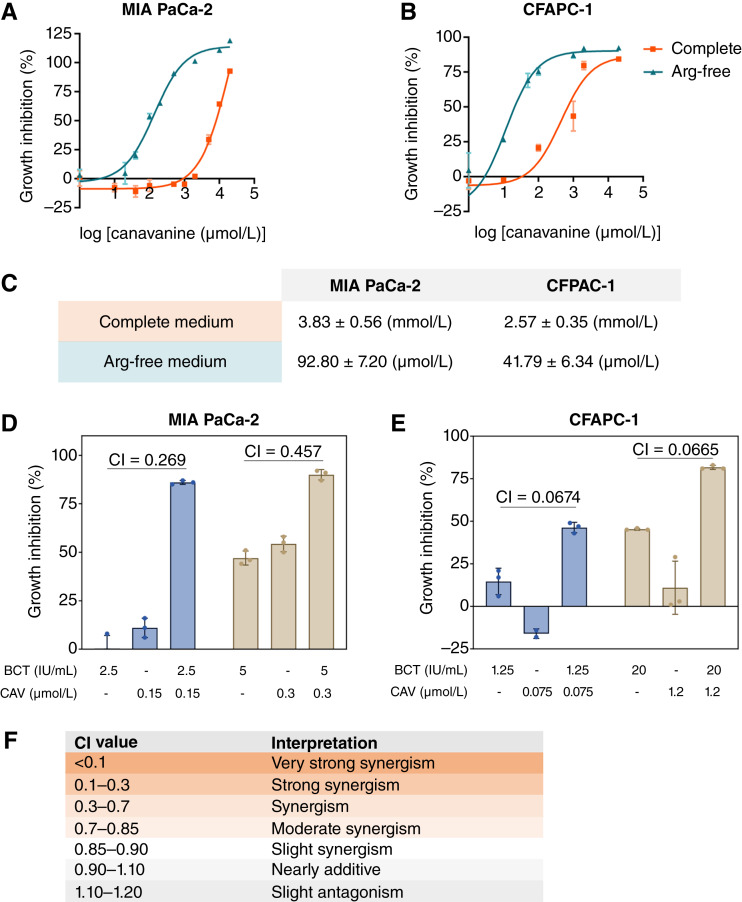
Arginine deprivation by PEG-BCT-100 synergizes with canavanine in pancreatic cancer cell growth inhibition. **A** and **B,** IC50 values of canavanine under arginine-rich conditions with complete medium and arginine-free medium were determined via cell viability assay after canavanine treatment. The dose–response curves shifted to the left under arginine depletion upon treatment. **C,** The IC50 values for MIA PaCa-2 and CFPAC-1 cells in complete medium were in the millimolar (mmol/L) range, whereas under arginine-free conditions, the values were in the micromolar (μmol/L) range. **D** and **E,** MIA PaCa-2 and CFPAC-1 cells were treated with a constant ratio of PEG-BCT-100 to canavanine for 48 hours, and cell viability assay was performed. Representative dosage combinations were presented, and the combination index (CI) was calculated using CalcuSyn software. **F,** The degrees of synergism were classified by CI values. Synergistic, additive, and antagonistic effects are defined by a CI value that is smaller than 1.0, equal to 1.0, or larger than 1.0, respectively. Arg-free, arginine-free; BCT, PEG-BCT-100; CAV, canavanine.

The pancreatic cancer cell lines MIA PaCa-2 and CFPAC-1 were treated with canavanine over a broad range of concentrations (1–20000 μmol/L). The treatment was carried out under two different conditions, namely, in complete medium with sufficient arginine or in arginine-free medium, and the cytotoxicity of canavanine was evaluated (Fig. 1A–C). The IC_50_ values of canavanine, when tested under arginine-sufficient conditions in complete medium, were in the millimolar (mmol/L) range. However, when arginine was removed by using an arginine-free medium, the IC_50_ values significantly decreased to the micromolar (μmol/L) range, suggesting that depriving the cells of arginine enhances the effectiveness of canavanine (Fig. 1D). To consolidate the findings, a constant ratio of PEG-BCT-100 to canavanine were administered to the cells, and the analysis of drug interactions was determined using the combination index calculated with CalcuSyn (23). Representative dosage combinations were presented, indicating that a synergism was observed between PEG-BCT-100 and canavanine in inhibiting the growth of pancreatic cancer cell lines (Fig. 1E and F).

### PEG-BCT-100 and canavanine induced apoptosis in pancreatic cancer cells

**Figure 2:**
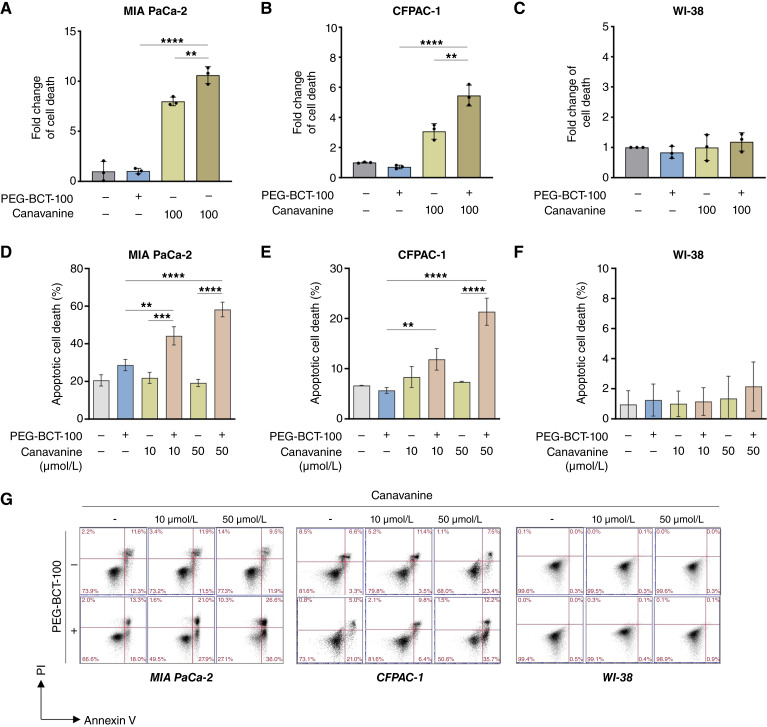
PEG-BCT-100 and canavanine induced apoptosis in pancreatic cancer cells. **A–C,** The effect of PEG-BCT-100 (0.3 IU/mL), canavanine (100 μmol/L), or their combination on cell death in pancreatic cancer cells (MIA PaCa-2 and CFPAC-1) and normal fibroblasts (WI-38) was quantified using the cell death ELISA assay. **D–F,** Annexin V and PI costaining was performed following treatment with canavanine (10 or 50 μmol/L), PEG-BCT-100 (0.3 IU/mL), or their combination. Apoptotic cell death was quantified, including both early- and late-phase apoptosis. **G,** Representative dot plots from annexin V and PI costaining flow cytometry analysis were shown. Bottom left quadrant, viable cells; bottom right quadrant, early apoptotic cells; and top right quadrant, late apoptotic cells. **, *P* < 0.01; ***, *P* < 0.001; ****, *P* < 0.0001.

We further investigated whether the observed cell growth inhibition was due to cell-cycle arrest or the induction of cell death. Apoptotic DNA fragments were detected using the cell death ELISA assay in MIA PaCa-2 and CFPAC-1 cells upon combination drug treatment but not in the WI-38 normal fibroblast cells (Fig. 2A–C). Notably, the levels of apoptotic events in the cotreated pancreatic cancer cells were considerably higher than those observed in the individual treatments with PEG-BCT-100 or canavanine alone (Fig. 2A and B). To substantiate our findings, we conducted annexin V/PI costaining using flow cytometry. The percentage of pancreatic cell lines undergoing early and late apoptosis was significantly increased in a dose-dependent manner with the combination treatment (Fig. 2D and E). However, this effect was not observed in the WI-38 fibroblasts, with representative plots from flow cytometry analysis shown (Fig. 2F and G). The time-course study involved the detection of apoptotic protein markers, namely, cleaved PARP and cleaved caspase-7 and caspase-9. The responses of the tested cell lines were observed to be optimal at distinct time points (Supplementary Fig. S1A).

### Low-dose canavanine combined with PEG-BCT-100 minimizes the toxicity to normal cells

**Figure 3:**
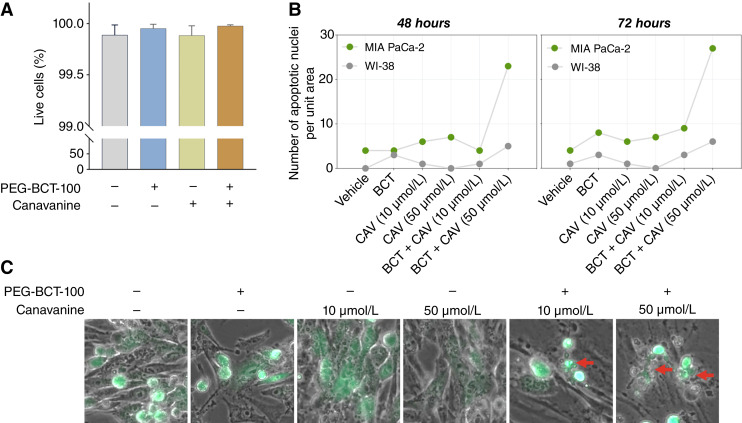
PEG-BCT-100 and canavanine induced apoptosis in pancreatic cancer cells. **A,** Live/dead staining was performed in normal fibroblasts, WI-38, following treatment with PEG-BCT-100 (0.3 IU/mL), canavanine (10 μmol/L), and their combination. The percentage of live cells was quantified by fluorescent microscopy analysis. **B,** GFP-expressing MIA PaCa-2 stable cells and fibroblasts (WI-38) were cocultured in a live cell chamber, and time-lapse experiment was conducted for 72 hours with images captured at 2-hour intervals. The number of apoptotic nuclei per unit area was quantified by fluorescent microscopy analysis after 48- and 72-hour drug treatment. **C,** Representative images at the final time point showed that the GFP MIA PaCa-2 cells were fragmented into small apoptotic bodies, indicated by the red arrows, when subjected to the combined treatments. BCT, PEG-BCT-100; CAV, canavanine.

Based on the aforementioned findings, we observed that with low-dose canavanine (50 or 10 μmol/L), which is approximately half and one-fifth of the IC_50_ value of pancreatic cancer cell lines, the induction of apoptosis in WI-38 fibroblast cells was minimal. Furthermore, we conducted live/dead staining after cotreatment with PEG-BCT-100 and canavanine and revealed that the viability of WI-38 cells remained unaltered (Fig. 3A). To examine the specificity of drug response, coculture experiment was performed using WI-38 fibroblasts and GFP-expressing stable cell line of MIA PaCa-2, with live-cell imaging monitored for 72 hours. Notably, the cotreated GFP MIA PaCa-2 cells started fragmenting into apoptotic bodies at 48 hours. In contrast, the WI-38 fibroblasts remained intact, and the apoptotic nuclei were merely detected throughout the coculture (Fig. 3B and C; Supplementary Fig. S1B). This indicates that a relatively low dose of canavanine in the presence of PEG-BCT-100 was sufficient in causing cell death in pancreatic cancer cells while minimizing the cytotoxicity toward normal cells.

### Low OTC and ASS1 expression levels facilitates the response to combined treatment

**Figure 4:**
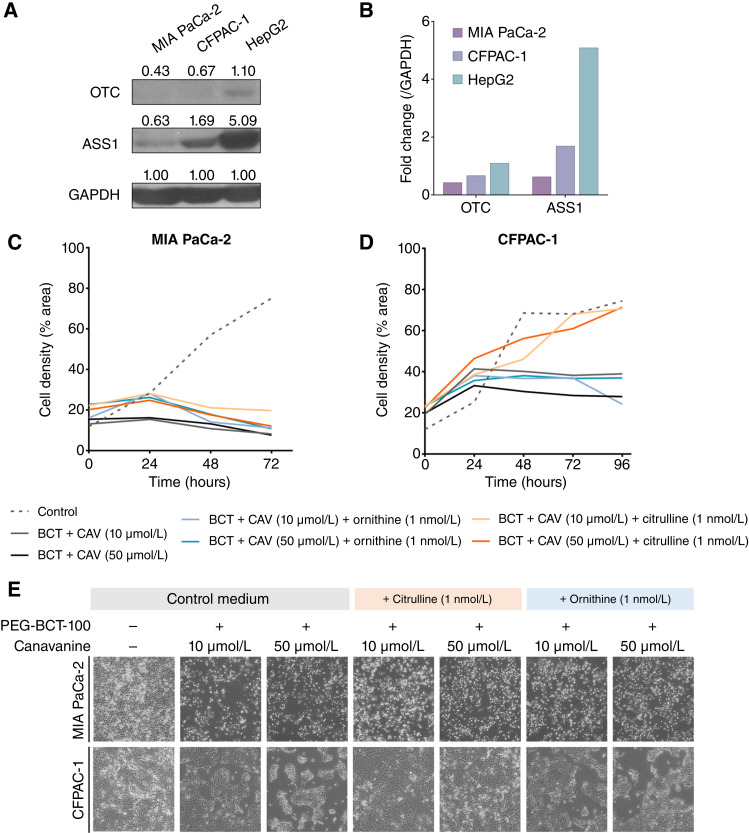
Low expression of OTC and ASS1 facilitates the response to the combined treatment. **A,** Western blot was performed to examine the basal expression level of ASS1 and OTC in the pancreatic cancer cells (MIA PaCa-2 and CFPAC-1) with HepG2 as a positive control. **B,** The endogenous expression level of ASS1 and OTC was quantified and normalized to GAPDH. **C** and **D,** In the rescue experiment, the cells were supplemented with urea cycle substrates, namely, ornithine (OTC substrate) and citrulline (ASS1 substrate), during the combined treatment. The cell growth rate was evaluated by measuring the cell density at different time points. **E,** Representative images at the final time point demonstrated the sensitivity to the combined treatment compared with the treatment without any supplementation. BCT, PEG-BCT-100; CAV, canavanine.

The expression levels of enzymes involved in the *de novo* biosynthesis of arginine were examined in the pancreatic cancer cell lines. OTC was undetectable in both MIA PaCa-2 and CFPAC-1 cells. MIA PaCa-2 showed low expression of ASS1, whereas CFPAC-1 had a relatively high protein level with HepG2 as a positive control (Fig. 4A and B). In order to validate the role of these enzymes in drug response, we conducted a proof-of-concept rescue experiment by supplementing the substrate of the respective enzymes during drug treatment. In the urea cycle of arginine synthesis, ornithine is the substrate for OTC, whereas citrulline is the product of OTC and substrate for ASS1 (24). MIA PaCa-2 cells exhibited higher susceptibility to the cotreatment, as indicated by the drastic reduction in cell density following the administration of PEG-BCT-100 and canavanine. The deficiency of both OTC and ASS1 enzymes in MIA PaCa-2 cells hindered the restoration of cell growth, even with the addition of citrulline or ornithine (Fig. 4C and E; Supplementary Fig. S2A). In the case of OTC^-^ASS1^+^ CFPAC-1 cells, cell growth was also not recovered with the addition of the OTC substrate, ornithine. However, with an abundant supply of citrulline, which serves as a substrate for ASS1 in regenerating arginine, the confluency of cells was well-restored, even under high-dose canavanine (50 μmol/L) in the presence of PEG-BCT-100, as shown in the representative images (Fig. 4D and E; Supplementary Fig. S2B). These results demonstrated that low expression of OTC and ASS1 facilitates arginine deprivation, thereby enhancing the efficacy of canavanine.

### PEG-BCT-100 enhances the antitumor effect of canavanine *in vivo*

**Figure 5:**
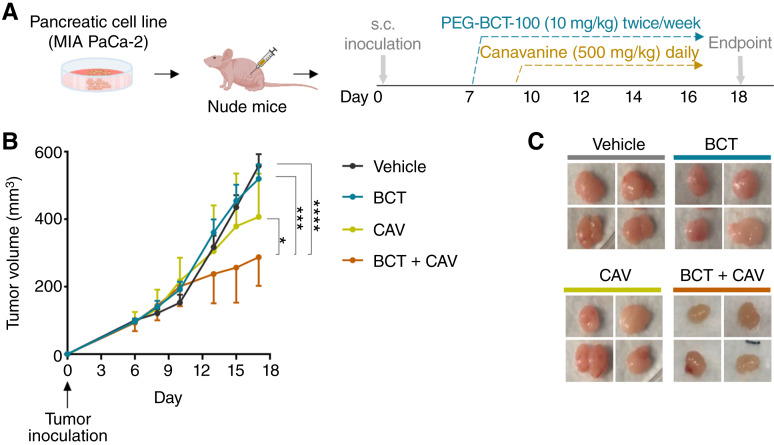
PEG-BCT-100 enhances the antitumor effect of canavanine *in vivo*. **A,** Nude mice bearing MIA PaCa-2 xenografts were grouped into vehicle control (*n* = 9), 10 mg/kg PEG-BCT-100 (*n* = 10), 500 mg/kg canavanine (*n* = 8), or combined therapy (*n* = 10) groups with the treatment schedule as shown. **B,** Tumor growth curve was determined by measuring tumor volumes using a caliper. **C,** Representative images showing the tumor morphology when the mice were sacrificed. *, *P* < 0.05; ***, *P* < 0.001; ****, *P* < 0.0001.

Given the promising results of the combination treatment *in vitro*, we established the MIA PaCa-2 xenograft model to evaluate the efficacy *in vivo*. The mice were divided into four groups and administered either the vehicle, PEG-BCT-100, canavanine, or the combination treatment (Fig. 5A). Considering the mechanistic rationale involving PEG-BCT-100’s role in arginine depletion and canavanine’s function as a toxic analog, PEG-BCT-100 was first delivered to the mice 2 days prior to the canavanine treatment. The combination of PEG-BCT-100 and canavanine exhibited the most pronounced antitumor effect on MIA PaCa-2 xenografts compared with the individual treatments (Fig. 5B and C). There was a slight weight loss of approximately 10% in the combinational treatment group (Supplementary Fig. S2C). Overall, the synergistic action of PEG-BCT-100 and canavanine not only suppressed tumor growth *in vitro* but also remained effective with the *in vivo* findings.

### Low expression of OTC and ASS1 in human pancreatic cancers

**Figure 6:**
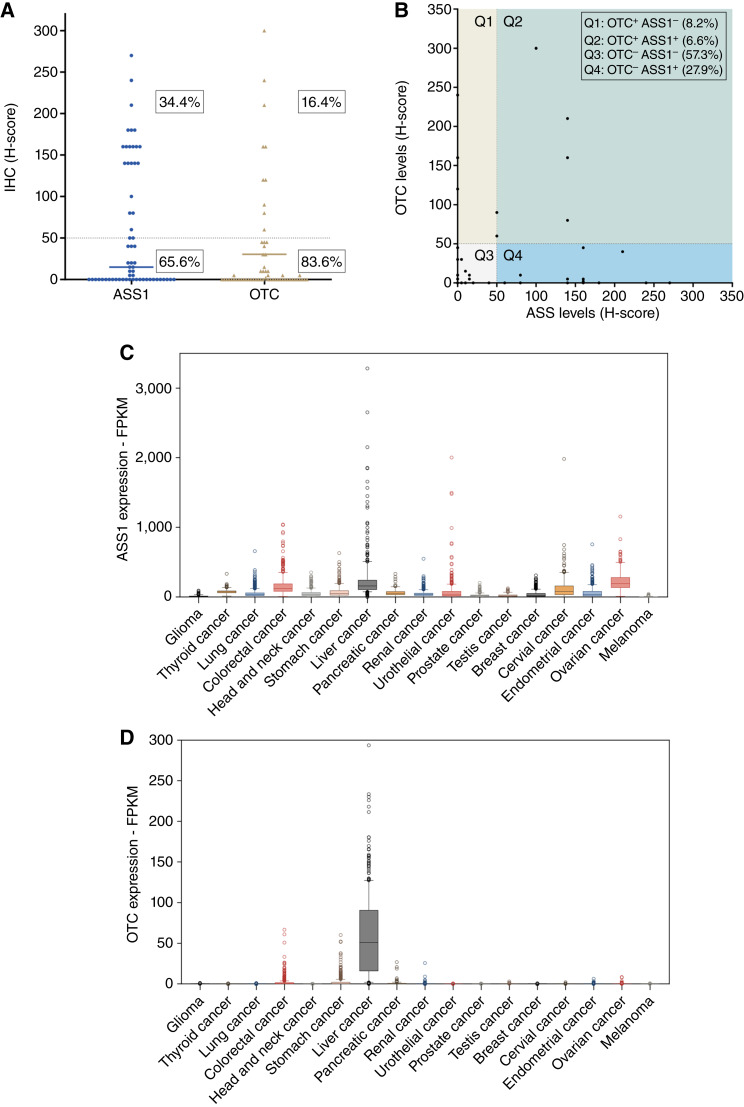
Low expression of OTC and ASS1 in human pancreatic cancers. **A,** The protein levels of ASS1 and OTC were quantified using the H-score method, in which the pathologist assessed the intensity and percentage of IHC staining in 61 pancreatic cancer tissue specimens. The optimal cut-off value for H-score was determined by the pathologist, in which low expression levels were associated with cases showing negative or weak positive signals. **B,** The distribution of both ASS1 and OTC expression levels in individual patients was illustrated in the scatter plot. **C** and **D,** Pan-cancer analysis of gene expression for ASS1 and OTC was conducted using the dataset from TCGA.

As low ASS1 and OTC expressions were shown to facilitate the response to the combination treatment, we then investigated the clinical relevance of this therapeutic approach by analyzing our in-house patient cohort with pancreatic cancer. IHC staining was performed on 61 pancreatic cancer specimens, revealing that a majority of patients exhibited low or no expression of ASS1 and OTC enzymes. Specifically, according to the H-score determined by the pathologist, 65.6% of the patients exhibited a low level of ASS1, whereas 83.6% of the patients showed a low level of OTC expression (Fig. 6A). Notably, 57.3% of the patients lacked both OTC and ASS1 enzymes, indicating most of the pancreatic cancers are arginine-auxotrophic (Fig. 6B). We extended our analysis to the TCGA RNA sequencing dataset, which includes more than 170 human pancreatic adenocarcinoma cases. In line with our own cohort, pancreatic cancers showed minimal expression of both OTC and ASS1 compared with other cancer types (Fig. 6C and D). This observation further supports the use of PEG-BCT-100 and canavanine, along with OTC and ASS1 as biomarkers, for predicting the treatment response.

## Discussion

**Figure 7:**
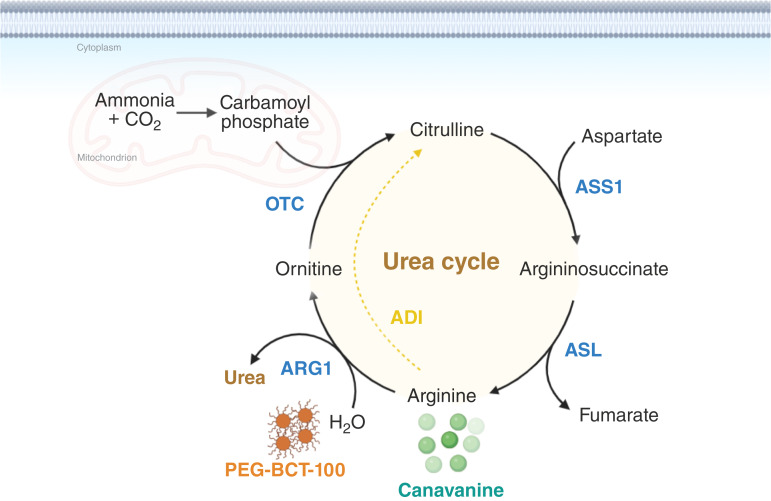
A working model involves the combination treatment of PEG-BCT-100 and canavanine. PEG-BCT-100 functions as an arginase, depleting arginine, enhancing the absorption of toxic canavanine, and resulting in synergistic antitumorigenic effects in pancreatic cancer cells. ADI, arginine deiminase; ASL, argininosuccinate lyase; ARG1, arginase 1.

Arginine deprivation is being explored as a promising anticancer therapy, with recent advancements overcoming the limitations of arginine-degrading enzymes by extending their half-life through PEGylation, which has been proven to be safe and effective in clinical trials (25). Arginine deprivation by PEGylated arginase has been shown to trigger cell-cycle arrest in hepatocellular carcinoma and T-cell acute lymphoblastic leukemia (26, 27). Despite its capability in inducing cell-cycle arrest, the cell death effect was not satisfactory when arginase was used as a single-agent therapy. On the other hand, canavanine, a toxic analog of arginine, has faced challenges in its development as a standalone therapeutic agent due to its ineffectiveness and general toxicity *in vivo*. Interestingly, canavanine has shown selective uptake by the pancreas when circulating in the body, providing further support for its potential application as a therapeutic supplement in pancreatic cancer (28). In this study, we combined PEG-BCT-100 and canavanine to achieve a maximal therapeutic outcome. The continuous depletion of arginine favors the uptake of canavanine by the cells, particularly in arginine-auxotrophic cancer cells (Fig. 7).

We observed that the inhibition of cancer cell growth by canavanine was limited under complete medium condition due to the competition with arginine in the extracellular environment. The cells tolerated canavanine well with concentrations in the millimolar (mmol/L) range, which is not considered therapeutically relevant, suggesting that canavanine alone may not be suitable as an antitumor agent. However, under arginine-deficient conditions, the IC_50_ value of canavanine was significantly decreased to the micromolar (μmol/L) range. Our results demonstrated that the combined treatment induced programmed cell death in MIA PaCa-2 and CFPAC-1 cells. This apoptotic effect could possibly be attributed to the prolonged autophagy triggered by arginine starvation (29). Moreover, the substitution of arginine by canavanine in the biosynthesis of proteins resulted in metabolic disruption, ultimately culminating in cell death (30). Collectively, our findings supported the notion that PEG-BCT-100 inhibits cancer cell proliferation and synergizes with canavanine to induce cell death.

The combination approach exhibited cytotoxicity specifically in pancreatic cancer cells, whereas normal fibroblasts (WI-38) remained unaffected. Canavanine was administered at concentrations approximately half and one-fifth of the IC_50_ values for pancreatic cancer lines, yet the survival of WI-38 cells was unaltered. Owing to the synergistic effect mentioned above, a relatively low dosage of canavanine was sufficient to induce cell death in cancer cells while minimizing gross toxicity to normal cells. The observed selectivity can be attributed to the metabolic reprogramming of tumor cells, which necessitates additional nutrients for their high energy demand and rapid growth (31–33). As endogenous nutrient sources become insufficient, tumor cells rely on exogenous sources. In contrast, normal cells can meet their energy requirements through endogenous catabolism to maintain homeostasis under normal conditions. In addition, WI-38 cells exhibited modest OTC expression, whereas MIA PaCa-2 and CFPAC-1 cells demonstrated a lack of OTC enzyme activity (Supplementary Fig. S3). This suggests that arginine could potentially be resynthesized through OTC in WI-38 cells, rendering them unaffected by the combined drug treatment. This significant disparity between normal and malignant cells can be exploited to selectively eliminate tumor cells, as demonstrated in our coculture experiment. It has been noted that normal cells were quiescent upon arginine deprivation and recovered after reintroducing arginine, further confirming the specificity of the combined treatment to pancreatic cancer cells (34, 35).

Arginine-auxotrophic cancers are known to exhibit greater sensitivity to arginine deprivation. Tumors lacking both OTC and ASS1 expressions are expected to be highly responsive to the combined treatment. This was confirmed in our rescue experiment in which double-negative MIA PaCa-2 cells showed significant cell growth inhibition, indicating their inability to recycle ornithine or citrulline into arginine. We questioned whether the cotreatment would remain effective for OTC^-^ASS1^+^ CFPAC-1 cells. Remarkably, the growth inhibition effect persisted in the presence of ornithine (OTC substrate), indicating that the combined therapy is still effective for OTC-negative cells regardless of ASS1 expression. In the urea cycle, the enzymatic reaction of ASS1 occurs after OTC, using citrulline (the product of OTC catalysis) as its substrate. Therefore, supplementing citrulline in CFPAC-1 cells mimicked the presence of OTC enzyme as well, allowing the ASS1^+^ cells to regenerate arginine and experience rescue in terms of cell growth. Overall, OTC serves as a more reliable predictive biomarker for the response to the combined treatment. Our findings also indicated that OTC deficiency is more prevalent than ASS1 in human pancreatic cancer.

It is noteworthy that the effectiveness of this cotreatment in both ASS1^−^ and ASS1^+^ pancreatic cancer cells is unique to the arginine depletion imposed by PEG-BCT-100 but may not apply to other arginine-depleting agents. Previous studies using arginine deiminase have shown reduced efficacy in ASS1^+^ cells (36). This is because citrulline, the end product of arginine deiminase, can be utilized by the ASS1 enzyme as a substrate to regenerate arginine. In contrast, the breakdown product of PEG-BCT-100 is ornithine, which hinders arginine recycling in OTC-negative cells. Consequently, once the cells are deficient in OTC expression, the combined treatment with PEG-BCT-100 and canavanine is still potent regardless of the ASS1 expression.

In conclusion, our data suggested that the combination of arginine deprivation using PEG-BCT-100 and canavanine exerted a significant synergistic anticancer effect in pancreatic cancer both *in vitro* and *in vivo*. The deficiency in OTC and ASS1 in human pancreatic cancer facilitates this combination approach, which also serves as a predictive biomarker for the response to this novel therapeutic strategy.

## Supplementary Material

FigS1 Supplementary Figure 1

FigS2 Supplementary Figure 2

FigS3 Supplementary Figure 3
