# Supplementary figures and images for: PEG-BCT-100 and Canavanine Synergistically Induce Apoptosis in Arginine Biosynthetic Enzyme-Deficient Pancreatic Cancer
Source: Cancer Res Commun. 2024 Dec 23;4(12):3180–9. doi: 10.1158/2767-9764.CRC-24-0425 (PMC11695075; doi:10.1158/2767-9764.CRC-24-0425)

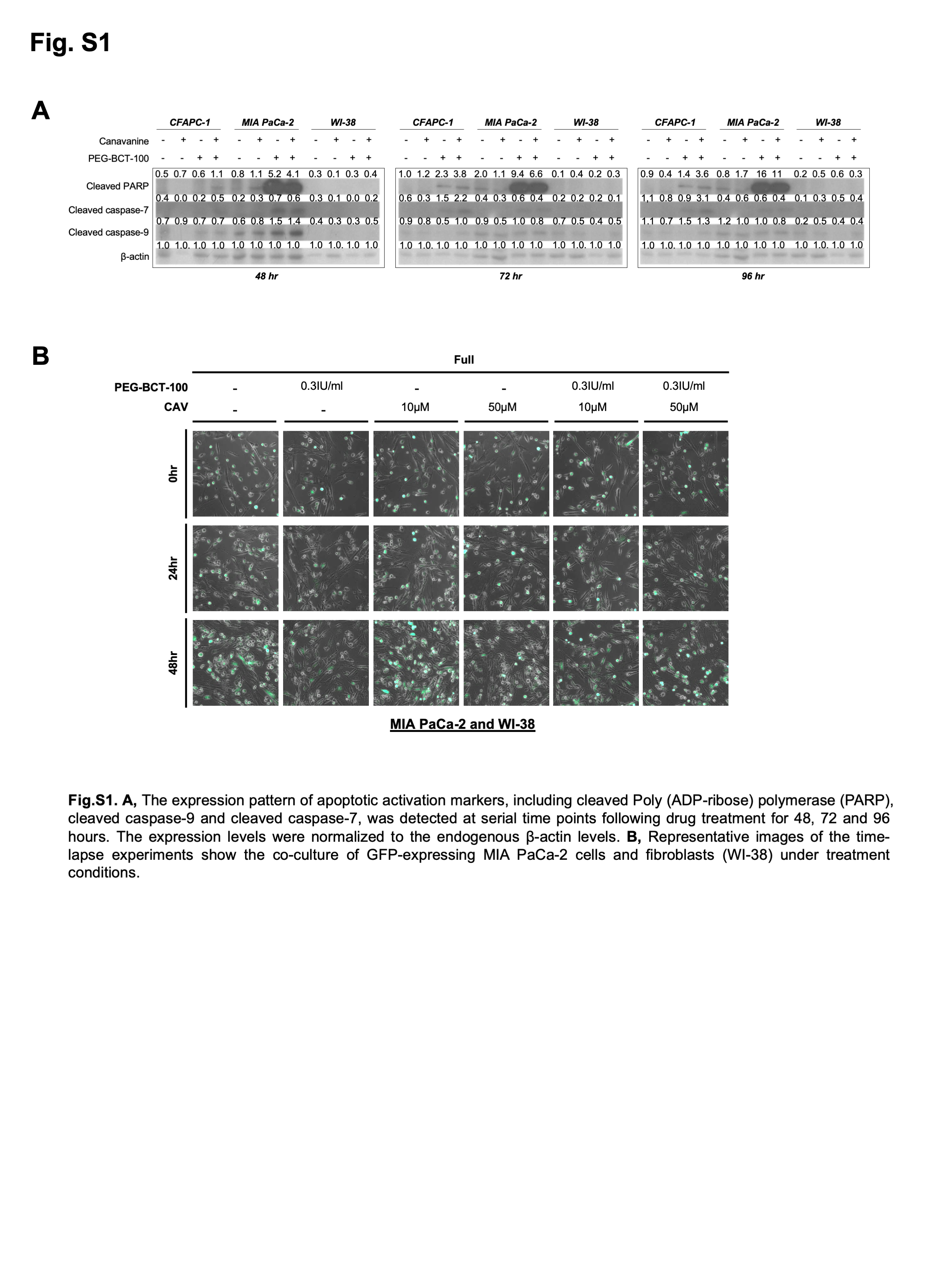

Supplement: FigS1 — Supplementary Figure 1 [file crc-24-0425_figs1_suppsf1.png]

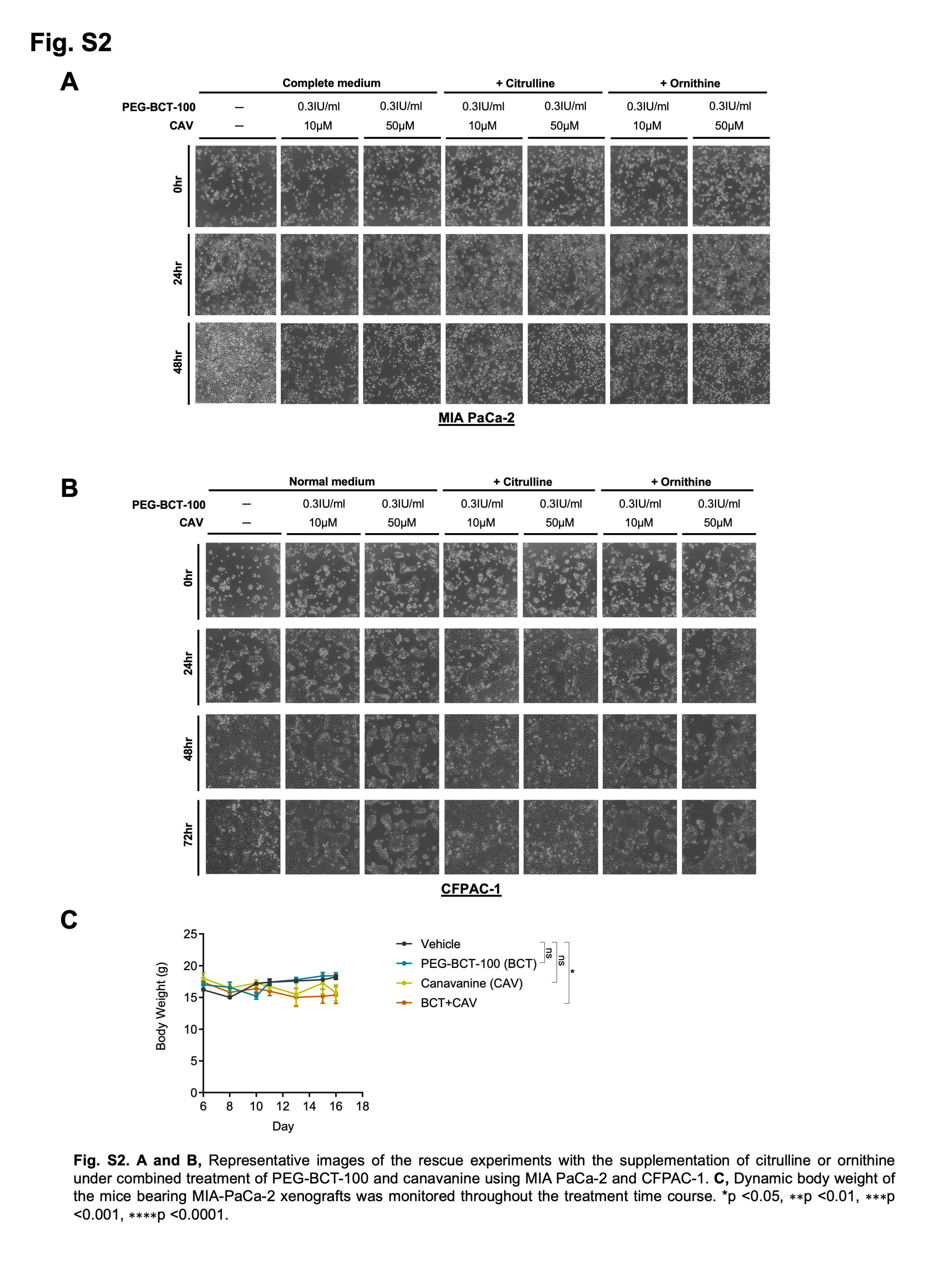

Supplement: FigS2 — Supplementary Figure 2 [file crc-24-0425_figs2_suppsf2.png]

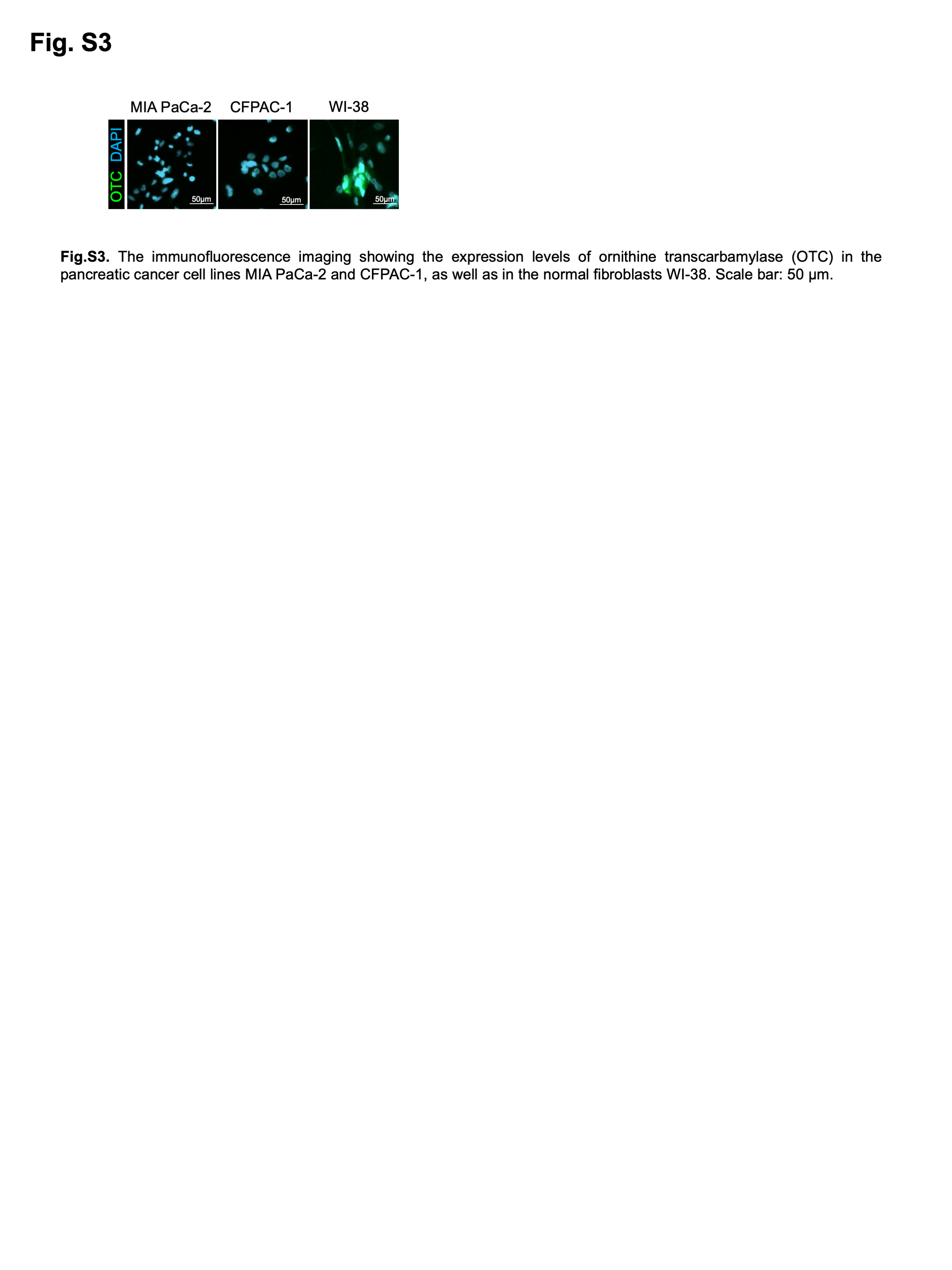

Supplement: FigS3 — Supplementary Figure 3 [file crc-24-0425_figs3_suppsf3.png]
